# First Evidence for Mechanism of Inverse Ripening from In-situ TEM and Phase-Field Study of *δ*′ Precipitation in an Al-Li Alloy

**DOI:** 10.1038/s41598-019-40685-5

**Published:** 2019-03-08

**Authors:** Jiwon Park, Reza Darvishi Kamachali, Sung-Dae Kim, Su-Hyeon Kim, Chang-Seok Oh, Christian Schwarze, Ingo Steinbach

**Affiliations:** 1 Korea Institute of Materials Science, 797 Changwondaero, Changwon, 51508 Republic of Korea; 2 Max-Planck-Institut für Eisenforschung, Max-Planck-Straße 1, 40237 Düsseldorf, Germany; 3 Interdisciplinary Centre for Advanced Materials Simulation (ICAMS), Ruhr-Universität Bochum, Universitätsstraße 150, 44801 Bochum, Germany

## Abstract

*In-situ* TEM investigation of aging response in an Al–7.8 at.% Li was performed at 200 °C up to 13 hours. Semi-spherical *δ*′ precipitates growing up to an average radius of 7.5 nm were observed. The size and number of individual precipitates were recorded over time and compared to large-scale phase-field simulations without and with a chemo-mechanical coupling effect, that is, concentration dependence of the elastic constants of the matrix solid solution phase. This type of coupling was recently reported in theoretical studies leading to an inverse ripening process where smaller precipitates grew at the expense of larger ones. Considering this chemo-mechanical coupling effect, the temporal evolution of number density, average radius, and size distribution of the precipitates observed in the *in-situ* experiment were explained. The results indicate that the mechanism of inverse ripening can be active in this case. Formation of dislocations and precipitate-free zones are discussed as possible disturbances to the chemo-mechanical coupling effect and consequent inverse ripening process.

## Introduction

Precipitate hardening is the main strengthening mechanism in Al alloys and has applications in the automotive and aerospace industries^1,2^. L1_2_ *δ*′ precipitate is one of the major hardening phases in low density Li-containing Al alloys^3^. The *δ*′ precipitate has a semi-stoichiometric composition (Al_3_Li) and holds a relatively small lattice misfit against the face-centred cubic (FCC) *α*-Al matrix^4^. Because of this small misfit and the almost spherical shape of the precipitate as well as the low temperatures needed for precipitation heat treatment, the binary Al-Li alloy was assumed as an excellent model system to study Ostwald ripening based on the Lifshitz-Slyozov-Wagner (LSW) theory^5,6^. Rylands^7^ conducted a comprehensive survey of the various studies conducted on different alloy compositions. Despite the fact that an overwhelming number of investigations reporting the classical ripening scaling ${t}^{\frac{1}{n}}$ with *n* = 3 are in agreement with the LSW theory^5,6^, their finding regarding the size distribution of *δ*′ precipitates are controversial. This motivated a detailed study of precipitation with different alloy compositions, that is, different precipitate fractions, in the late 1990s by Nembach and co-workers^8^. They analyzed the size distribution based on a model proposed by Ardell^9^, which consider the effect of precipitate volume fraction on the size distribution in the later stages of ripening under purely diffusive conditions. The comparison between the model and experimental results suggests a drastic increase in the capillarity length with the precipitate volume fraction, i.e. with the nominal alloy composition^8^. This is, however, in contrast to the fact that the equilibrium compositions of the coexisting phases (the precipitate and matrix phase) are rather thermodynamic functions independent of the nominal composition of the alloy. Thus, it is not clear how the capillarity constant depends on the nominal composition if we consider chemical effects only. In a more recent study, Tsao *et al*.^10^ conducted a small-angle X-ray scattering (SAXS) study of an Al–9.7 at.% Li alloy aged at 180 °C where a transition from a negative-skewed to a positive-skewed size distribution was observed. Again, this observation could be explained by accounting for the coalescence of the precipitates^11^ rather than the effect of precipitate volume fraction on the size distribution (Adell’s model)^12^. A discrepancy, however, arises here as well because it is not clear how the ripening exponent *n* = 3 is maintained in the presence of the coalescence mechanism.

In 2011, Glicksman and coworkers reinvestigated this unsolved problem of the precipitate size distribution by applying their recently developed diffusion screening and concluded that “Experimental characterization of microstructure evolution in three binary Al-Li alloys provides a quantitative test of diffusion screening theory. […] These experiments show that the diffusion screening theory for late-stage phase coarsening yields accurate predictions of maximum size of particle and relative coarsening constant.”^13^. In this study, the agreement of experimental observations of the ripening exponent with the LSW theory, and the disagreement with the volume fraction dependence of precipitate size distributions were reconfirmed, which was resolved this time by introducing diffusion screening^14^ that accounts for the finite diffusive exchange among neighbouring precipitates. To test the latter assumption of finite diffusion screening, direct phase-field simulations may be an appropriate method of study. One may, however, also ask whether there are other mechanisms beyond pure diffusion which may explain the discrepancy between the theory and experimental results. A recent study presented by two of the current authors has introduced the concept of *strained equilibrium* around a self-stressed precipitate^15^. Here, a concentration dependence of the elastic constants leads to a solute depletion or enrichment around a self-stressed precipitate, depending on whether solute atoms strengthen or soften the matrix phase. In a follow-up study, Kamachali and Schwarze^16^ proposed that chemo-mechanical coupling can drastically change the course of ripening to an *inverse ripening*, in which a smaller *δ*′ precipitate can grow at the expense of a larger one. This theory then was applied to an ensemble of coherent *δ*′ precipitates^17^ growing from small nuclei where the inverse ripening was confirmed and a narrowed size distribution was observed in an Al–8 at.% Li alloy annealed at 200 °C. In the current study, in order to resolve some of the above-mentioned discrepancies with respect to the precipitate size distribution and to study the effect of chemo-mechanical coupling we conduct a combined *in-situ* transmission electron microscopy (TEM) study and phase-field simulations of *δ*′ precipitation in an Al–7.8 at.% Li model alloy. We use the experimental data to adopt uncertainties in the theoretical model and compare the results in terms of number density, average radius, and size distribution of the precipitates. The findings are discussed in the context of previous studies. Concurrent events such as generation of dislocations network and formation of precipitate free zones (PFZ) are discussed as well. In the following section we present a brief introduction of the chemo-mechanical coupling effect. Thereafter, the *in-situ* TEM observations are presented and discussed against phase-field simulation results and previous studies. The experimental and simulation set-up are briefly described in the Methods section. Supplementary Materials provide more details of the experimental investigation.

### Chemo-mechanical coupling model

The subject of chemo-mechanical coupling has been extensively explored in the last century, starting with the seminal contributions by Làrche and Cahn^18,19^ and Khachaturyan^20^ who considered the effect of alloying on lattice distortion. This concept has been successfully applied to solid state transformation revealing the significance of elastic interactions in several phenomena such as spinodal decomposition^21^, precipitation^22–24^ and very lately in adsorption of hydrogen in Pd and Pd–Au alloy^25^. Recent studies have pointed out that a different type of coupling (composition dependence of elastic coefficients) may play a role in precipitation processes^15,16^. In first-order approximation, this effect can be expressed by a linear expansion of elastic constants in composition:

1 $${C}^{ijkl}(c)={C}_{0}^{ijkl}(1+\kappa \,{\rm{\Delta }}c)$$

where ${C}_{0}^{ijkl}$ are the elastic constants of a reference composition *C*_ref_ and *κ* is the chemo-mechanical coupling factor. The composition variation ${\rm{\Delta }}c=c(x,t)-{c}_{{\rm{ref}}}$ is defined locally resulting in a spatial dependency of the elastic constants in the system. For the dilute Al-Li alloy considered in this study, ab-initio calculations confirm the linear composition dependence of elastic constants^17,26^. Once the coupling is considered, the flux of solute atoms does include a mechanically driven contribution:

2 $$\dot{c}=-\nabla \cdot {\bf{J}}=\nabla \cdot [M\nabla \frac{\delta F}{\delta c}]=\nabla \cdot [M(\frac{{\partial }^{2}{f}^{{\rm{chem}}}}{\partial {c}^{2}}\nabla c+\kappa \nabla {f}^{{\rm{elast}}}{|}_{c={c}_{{\rm{ref}}}})]\,\cdot $$

Here **J** is the solute flux, $F=\int [\,{f}^{{\rm{chem}}}+{f}^{{\rm{elast}}}]$ d*V* is the free energy functional of the material, *M* is atomic mobility, and *f* ^chem^ and *f* ^elast^ are chemical and elastic energy densities, respectively. While the first term in the parentheses corresponds to Fick’s laws, as per the second term, solute atoms diffuse from regions with higher elastic energy into regions with lower elastic energy. The significance of this mechanically driven subflux in the precipitation has been discussed in several previous works^15–17,27^. An analytical expression for the concentration profile has been derived to the first-order in the *κ* factor at equilibrium (${\bf{J}}=\overrightarrow{0}$) around a self-stressed precipitate suggesting equilibrium depletion or enrichment of solute content around the precipitate depending on the sign of the coupling factor (i.e., strained equilibrium)^15^. In case of a positive coupling factor (*κ* > 0), which means the increase in stiffness with increasing matrix concentration, it was shown that the solute atoms will be driven away from the precipitate to reduce the total elastic energy by reducing the local stiffness of the material. A numerical solution of this model was applied to studying the evolution of a single Ni_4_ Ti_3_ precipitate in a NiTi shape memory alloy^27^ where the strong Ni depletion close to the Ni_4_ Ti_3_ precipitate could be explained. Later, the concept of chemo-mechanical coupling was extended for studying a pair of *δ*′ precipitates in an Al–8 at.% Li alloy where an *inverse ripening* phenomenon was predicted and discussed^16^. Solving the problem for a quasi-steady state ($\dot{c}=0$) with mean-field boundary conditions showed that above a critical precipitate size a non-vanishing flux **J** will be established from the larger precipitate to the smaller one, which enforces inverse ripening of the precipitates. An application of the model to an ensemble of many *δ*′ precipitates in an Al–9 at.% Li alloy confirmed the inverse ripening phenomenon in a defect-free Al matrix^17^. The inverse ripening phenomena without chemo-mechanical coupling has been discussed in several previous studies. Johnson and coworkers^28,29^ proposed a mechanism of inverse ripening due to elastic interaction between a pair of precipitates without and with an external load. Wang *et al*.^30,31^. performed a phase-field study and showed that precipitates may undergo a local inverse ripening due to their elastic interactions, which strongly depend on the arrangement of the precipitates. Su and Voorhees^32,33^ showed that the occurrence of inverse ripening strongly depends on the morphology, arrangement and size of neighbouring precipitates. In case of *δ*′ precipitation, however, it was shown that elastic interaction without a chemo-mechanical coupling effect does not lead to an inverse ripening process^17^.

**Figure 1:**
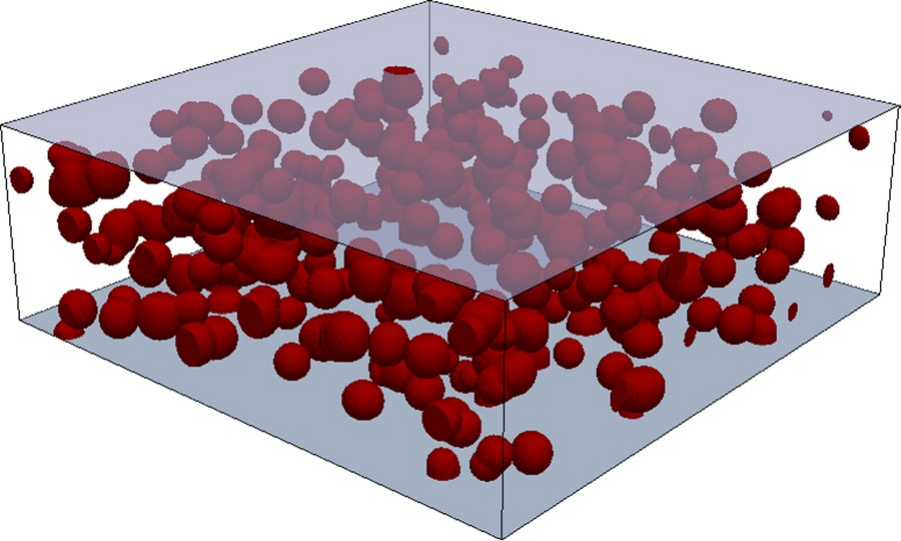
Initial setting of the simulation box is shown, constructed based on the size information extracted from the *in-situ* measurments. Precipitate sites are chosen randomly. Li sink layers were placed at the upper and lower surfaces (coloured blue) of the simulation box. The box size is 300 × 300 × 100 nm^3^.

In the current study, an *in-situ* TEM experiment was performed to investigate ripening of *δ*′ precipitates in an Al–7.84 at.% Li alloy. The results of the experiment were compared to large-scale phase-field simulations with and without the chemo-mechanical coupling effect. Figure 1 shows the initial set-up of the phase-field simulations that were set using the experimental measurements after 9 hours of *in-situ* aging. The coupling factor *κ* was adjusted to the experimental measurements as well. The effect of the microstructural defects on the ripening was also taken into account. In particular, a solute depletion associated with formation of dislocations was observed in the *in-situ* experiment, which was emulated by introducing a sink in the simulations (Fig. 1). The details of the *in-situ* experiment and simulation set-up are presented in the Methods section. Please also see Supplementary Information.

## Results and Discussion

**Figure 2:**
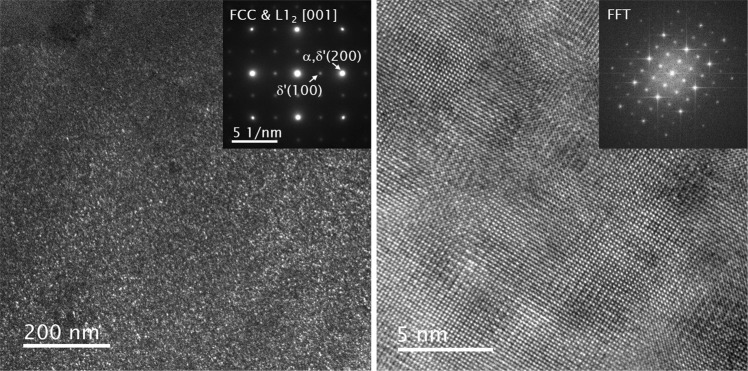
Dark-field and HR TEM images show the presence of the L1_2_ phase in an as-quenched specimen.

The presence of an ordered L1_2_ *δ*′ phase in as-quenched Al-Li alloys has been reported by a number of studies^34–38^ and was evidenced in the current study as well. Dark-field images corresponding to (100) diffraction in the L1_2_ and high resolution transmission electron microscopy (HRTEM) images in Fig. 2 clearly show homogeneously dispersed fine *δ*′ precipitates in an as-quenched specimen. Since the lattice parameter difference between *α*-Al and *δ*′ is small^39^, diffraction patterns from two phases overlapped and only {100} type diffractions from L1_2_ phase can be used to identify *δ*′ properly. Fourier-filtered HRTEM analysis from Fig. 2 shows that the size of *δ*′ in the as-quenched stage is less than 5 nm.

**Figure 3:**
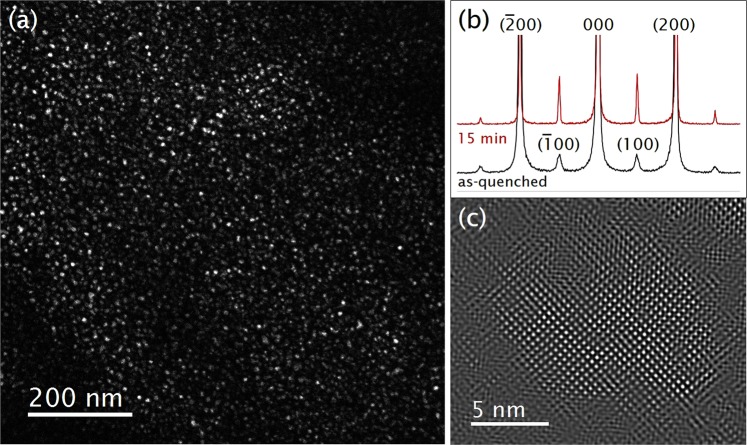
*δ*′ evolution after 15 minutes of aging is shown. (**a**) Dark-field image. (**b**) Line intensity profile of the diffraction patterns in [001] direction red: aged 15 minutes, black: as-quenched (**c**) Fourier-filtered image of *δ*′ precipitates.

Figure 3 shows the evolution of *δ*′ precipitates after 15 minutes *in-situ* aging at 200 °C. The development of {100} diffractions from the L1_2_ superstructure is evident (Fig. 3(b)), indicating that the population of the precipitates increased during the aging. The average precipitate radius determined by the Fourier-filtered HR image in Fig. 3(c) is approximately 5 nm. The uniform distribution of the precipitates indicates that the barrier for *δ*′ nucleation was small. This phenomenon has been often related to the nucleation assisted by the excess vacancies that are trapped during quenching; it is argued that in the presence of excess vacancies, the nucleation of the *δ*′ precipitate on the dislocations, which could be thermodynamically favourable, is kinetically hindered^40^.

**Figure 4:**
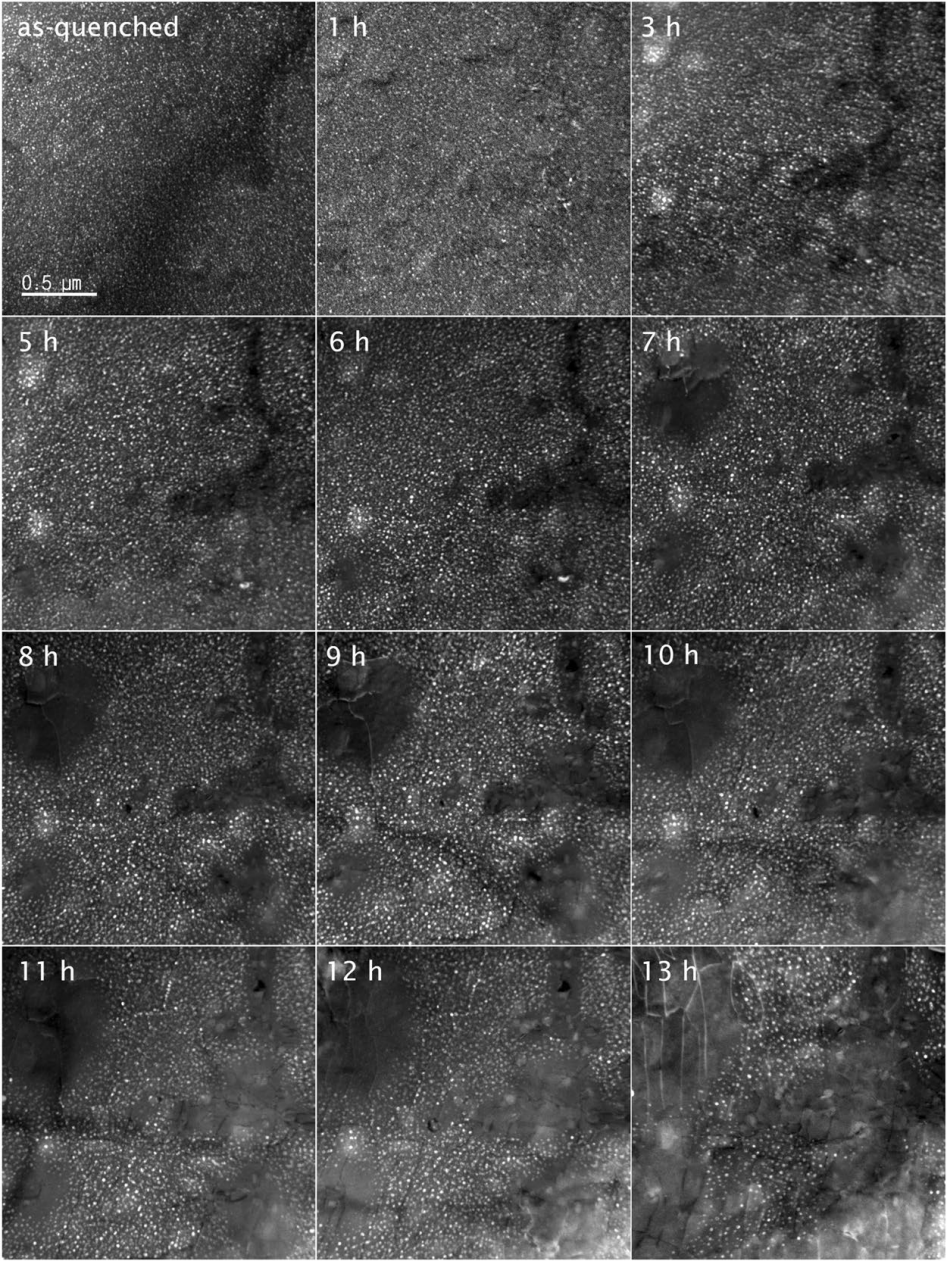
Dark-field images of *δ*′ (white particles) evolution during *in-situ* TEM observations are shown up to 13 hours.

Initially, the uniform small precipitates were observed to grow larger during the aging. The evolution of the precipitates up to 13 hours of aging is shown in Fig. 4. Since the existence of precipitates in an as-quenched specimen (Fig. 2) prevents the possibility of studying precipitation in the earlier stages of nucleation and growth using *in-situ* experiments, in this study, the results from the intermediate growth and ripening, that is, between 9 and 13 hours of aging, were used to validate against the simulation studies.

During the *in-situ* aging, strong developments in terms of dislocations and subsequent dissolution of *δ*′ precipitates were observed (see Fig. 4). The exact source of dislocations in the specimen is unclear; the pre-existing dislocations or roughness on the specimen’s surface can be possible dislocation sources. This is also likely that the cone shaped TEM specimen, very thin in the centre (with a hole produced during electrochemical thinning), was deformed as it expanded during the *in-situ* heating. Also, unlike the bulk material^40^, in our small sample there was no grain boundary for annihilation of dislocations or recrystallization. Hence the generated dislocations were forced to stay in the system. Once dislocations were generated in the matrix, complete dissolution of the precipitates, regardless of their size, was observed near the dislocations. Figure 4 shows the formation of precipitate-free microstructure after 7 hours of aging starting from the top-left and far right-side regions and expanding within the sample. Please also see the Supplementary Information for more details on dislocation generation.

The stress field around dislocations can strongly influence the chemo-mechanical coupling effect, which strictly depends on the stress state around the precipitates^15,16^. It is known that due to their larger radius, Li atoms can be attracted towards the dislocations to compensate the compressive stresses around the dislocations (formation of a Cottrell atmosphere-like^41^). Near the dislocations, the driving force for the segregation becomes larger than the chemical driving force for precipitation. Hence, precipitates shrink and the excess Li atoms segregate to the dislocation network. Baumann and Williams^42^ suggested that excess Li content released from dissolved *δ*′ precipitates can be stabilized in the vicinity of the dislocations (viz., PFZs), where precipitation is initially suppressed due to depletion of vacancies. In the later stage of ripening, larger *δ*′ precipitates have often been reported to decorate dislocations and the borders of PFZs^42^. This was not observed in the current *in-situ* study, which was limited to 13 hours of aging at 200 °C.

**Figure 5:**
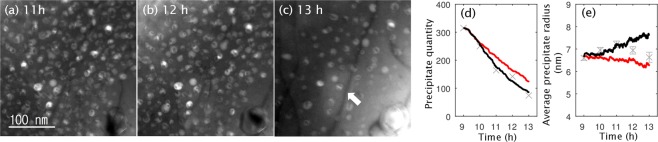
Dissolution of the precipitates after (**a**) 11 hours (**b**) 12 hours, and (**c**) 13 hours of aging is shown. The white arrow indicates a dislocation. The number density of the precipitate rapidly decreased between 12 and 13 hours. Evolution of (**d**) the precipitate quantity (number) and (**e**) average precipitate radius is presented and compared to the simulation results. Grey markers: experiments, lines: simulation without chemo-mechanical coupling, *κ* = 0, (black) and with chemo-mechanical coupling, *κ* = 0.05 at.%^−1^, (red). The grey bars indicate the standard error of measurement.

The number and size of the individual *δ*′ precipitates were measured during the *in-situ* experiment. Figure 5(a–c) show the decrease in the number of precipitates in a zoomed-in region of the specimen between 11 and 13 hours of aging. Even with a small number of dislocations in the visible region, the overall dissolution of the precipitates is evident indicating the dominant influence of a Li sink in the system. Beside dislocations, free surfaces of the specimen could be another sink for the Li atoms, where Li atoms can segregate to compensate for the surface stresses. Though it is not possible to identify all different sinks for Li, one can approximate the amount of Li loss at any given time by evaluating the volume fraction of the precipitates. This information was extracted and used to emulate the decreasing amount of Li in the phase-field simulations where a virtual Li sink was considered, as described in the following (see Methods section for technical details).

In order to compare the experimental results versus simulations, two parameters of simulations, i.e. the strength of Li sink and the chemo-mechanical coupling factor (*κ*) should be investigated. For this purpose, first we use the quantity (number) of precipitates to determine the strength of the Li sink such that it guarantees a match between both simulation cases (without and with a coupling effect) and the experimental observation. So we are left with only one fitting parameter (the chemo-mechanical coupling factor) to investigate the size evolution of the precipitates. The decline in the precipitates number can be not only due to the Li sink but also due to the conventional ripening. But since, at least next to the dislocations, precipitates were observed to disappear irrespective of their size, the current choice for the Li sink seems reasonable that gives the graphs presented in Figure 5(d). This will be further discussed in the following. The effect of chemo-mechanical coupling is then examined by studying average precipitate radius and precipitate size distribution for two cases where a positive chemo-mechanical coupling factor *κ* = 0.05 at.%^−1^ was compared to a simulation without the coupling *κ* = 0 at.%^−1^ (note that in the absence of chemo-mechanical coupling the usual elasticity is still active).

**Figure 6:**
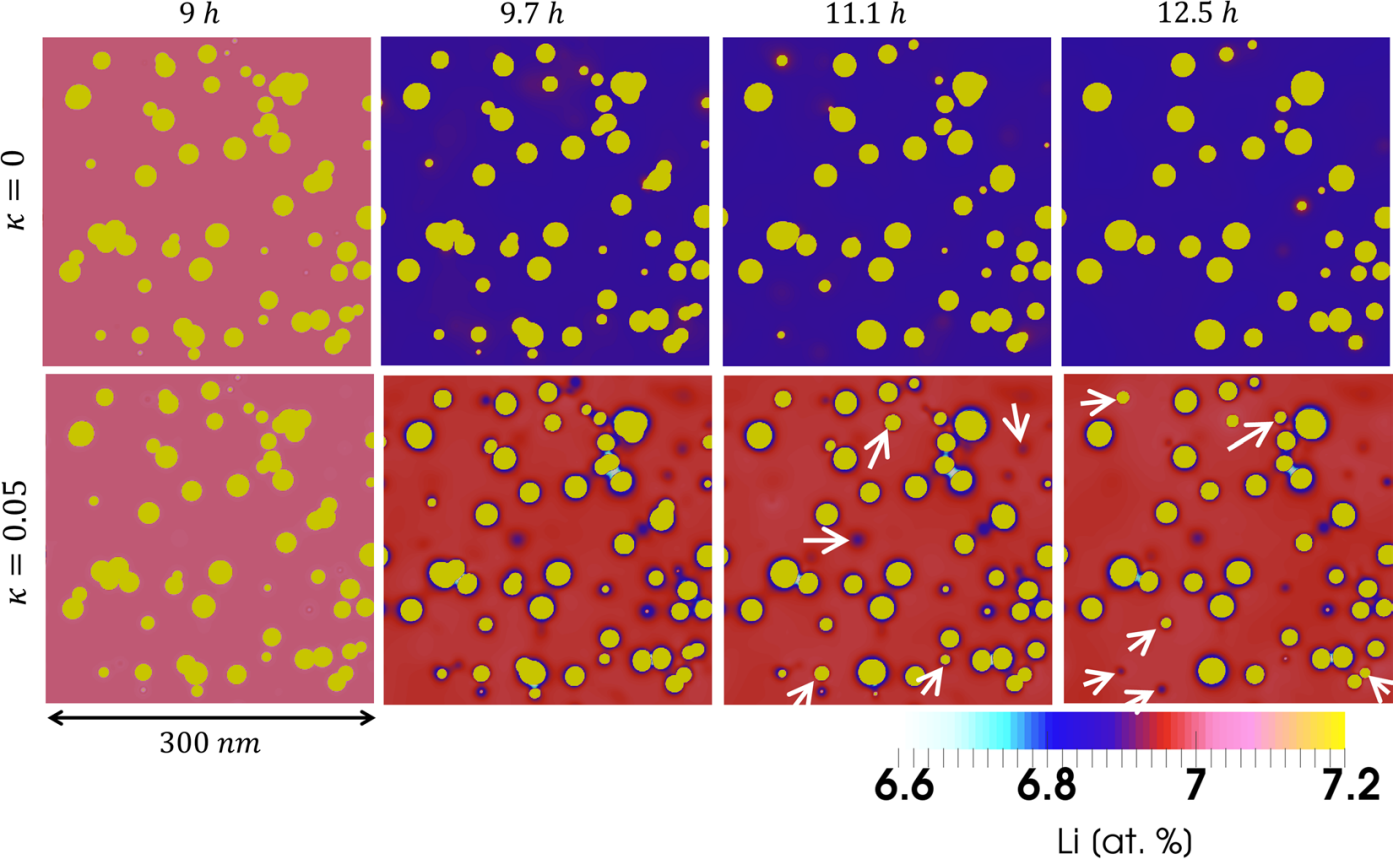
2D cross-sections of concentration fields inside the simulation boxes are shown over time. The colour bar applies for all concentration maps. The precipitates appears yellow and contain a constant 25 at.% Li. Strong depletion next to the precipitates and higher concentration in the matrix are characteristics of the chemo-mechanical effects (see also^16^) observed here. The white arrows point some precipitates which did not disappear in the presence of the chemo-mechanical effect. The initial overlap of some precipitates is due to the random initialization. The coalescence of these precipitates however is not allowed. See more details in the Supplementary Information and Method section.

Figure 5(d) shows the evolution of the precipitate quantity (number) over time from the *in-situ* experiments and phase-field simulations. (In the graphs, the solid lines represent phase-field simulations and the grey markers/bars represent experimental measurements. The black and red curves correspond to the phase-field simulations without and with the chemo-mechanical coupling effect, respectively.) The quantity of precipitates continuously reduces in the experiment as well as in the simulations. The results show that both simulation cases, with and without the chemo-mechanical coupling, reasonably reproduce the measured quantity of precipitates over time in the considered domain, which illustrates that the Li sink in the experimental set-up is very well modelled independent of the chemo-mechanical coupling effect. For the simulation case without a chemo-mechanical coupling this decrease can be due to the existence of Li sink as well as conventional ripening. Figure 5(e) shows the evolution of average precipitate radius. While the average precipitate radius decreased in the experiment after about 11 hours of aging, a monotonic increase is observed for the simulation without the chemo-mechanical coupling. This indicates that the conventional ripening is dominant in this simulation case, even in the presence of Li sink, when the chemo-mechanical coupling is not considered (*κ* = 0). In contrast, the average precipitates size in the presence of the chemo-mechanical coupling (*κ* = 0.05) shows a decrease that compares better to the experimental observations. Given that all inputs for both simulations are identical, the difference in the temporal evolution of the average precipitate radius is due to the chemo-mechanical coupling effect; When the precipitates are stabilized by an inverse ripening mechanism^16,17^, the conventional ripening will be suppressed, and the average precipitate radius has to eventually decrease in the presence of the Li sink (Fig. 5(e)), i.e. the precipitates lose their solute content to the sink. Figure 6 shows 2D concentration maps for both simulations without and with a chemo-mechanical coupling over time. Two characteristic features of the chemo-mechanical coupling^16^, i.e. higher concentration level in the matrix and strong solute depletion next to the precipitates, are observed in the current simulations as well. Several precipitates that disappeared during conventional ripening but remained in the presence of chemo-mechanical coupling are marked by white arrows in Fig. 6. It is to note that, the initial overlap of some precipitates is due to the random initialization procedure. Since, however, each precipitate is indexed with an unique phase-filed parameter, the coalesce of the precipitates is prohibited that minimizes the effect of this kind of interaction. More details including 2D phase-field maps and 3D snapshots of the simulations are presented in the Supplementary Information.

**Figure 7:**
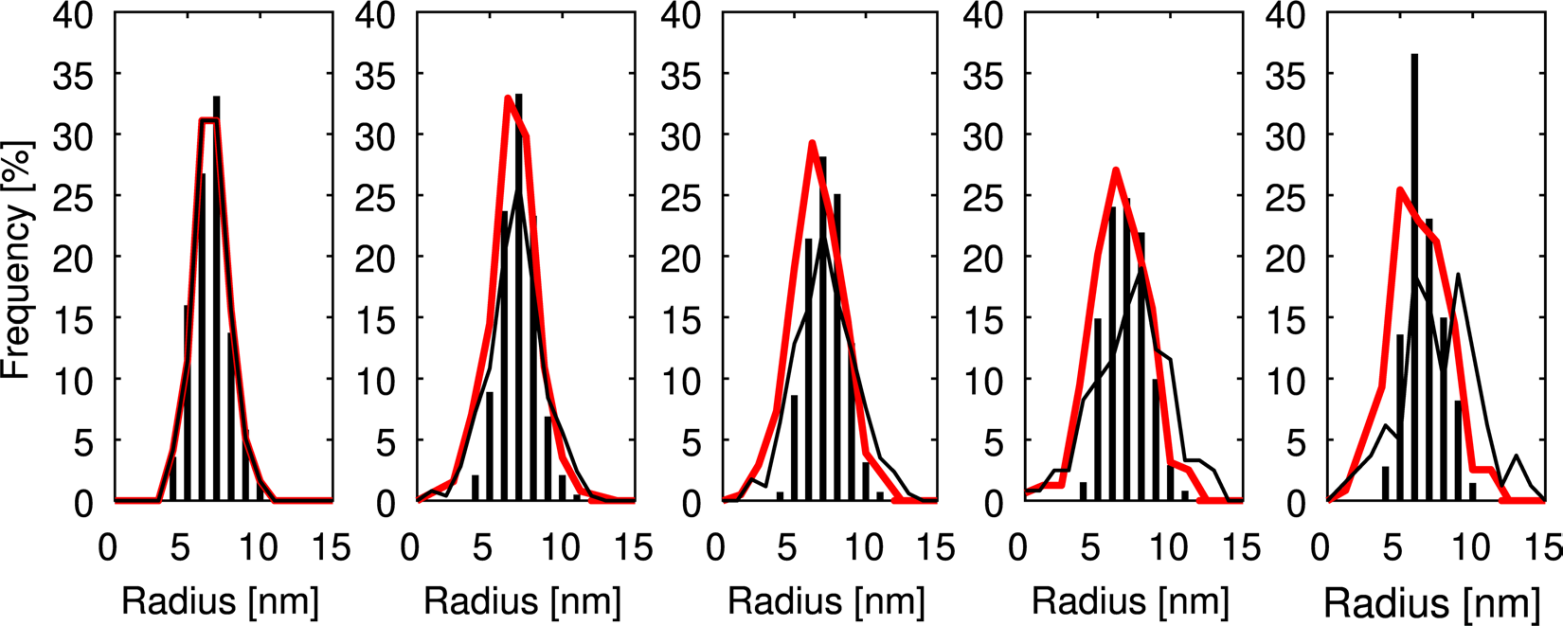
Precipitate size distribution at 9 h, 10 h, 11 h, 12 h and 13 h. Grey markers: experiments, lines: simulation without chemo-mechanical coupling, *κ* = 0, (black) and with chemo-mechanical coupling, *κ* = 0.05 at.%^−1^, (red).

As a second measure, the size distributions as a function of aging time can provide more detailed information about the evolution of the *δ*′ precipitates. In Fig. 7, the extracted data (absolute values of precipitate radii) from the *in-situ* experiment for 9, 10, 11, 12 and 13 hours are compared to the simulation results. The experimentally observed size distribution at 9 hours of aging was used to set the initial size distribution in the phase-field simulations. In the experiment, it was difficult to detect precipitates with a radius smaller than 3 nm which are not reported here, leading to a higher frequency of small precipitates (<3 nm) in the simulations compared to the experimental observations. It is observed that the size distributions in the *in-situ* experiments remain sharp with very limited broadening in the beginning. An initial broadening was observed in the simulations as well, with and without the chemo-mechanical coupling effect. However, with further evolution of the precipitates, broadening of the size distribution continued (black lines) when the chemo-mechanical coupling was not considered. Though the simulation results (in the absence of coupling) are consistent with the conventional theories of ripening, it is evident that these theories cannot simultaneously explain the experimental observations in terms of average radius and size distribution of the precipitates. This finding is also in agreement with the previous studies^8,10,13^, reviewed in the introduction section, which found disagreements between the experimentally observed size distributions and classical ripening theories. In the presence of the chemo-mechanical coupling (and the Li sink), instead, the size distribution remains finite and the precipitates shrink (dissolve) together and lose their Li content only to the sink, as the conventional ripening is inactive due to the inverse ripening. This causes a decrease in average precipitate radius as shown in Fig. 5(e). The narrow size distribution of the *δ*′ precipitates due to an inverse ripening mechanism has been discussed in previous theoretical studies^16,17^. In this case, the smaller precipitates maintain a larger driving force for growth and can grow at the expense of larger precipitates, establishing a narrow range of size (see Figs 3–5 in the theoretical paper^17^). The comparison between the simulation results and experiments indicates that the inverse ripening mechanism can be active, albeit for a short period of time. It is notable that here the spherical shape of *δ*′ precipitate helps the inverse ripening mechanism as in this case the elastic energy as well as interface energy, determined by the local curvature of the interface, are uniform in all directions allowing an isotropic chemo-mechanical interaction among the precipitates.

The inverse ripening phenomenon due to elastic interaction among the precipitates (without a chemo-mechanical coupling effect) has been previously reported in the literature. Johnson and coworkers^28,29^ and Mitazaki *et al*.^43^ have shown that under certain conditions, neighbouring precipitates might stabilize each other against coarsening, which is driven by a reduction in the total elastic energy of the system. Su and Voorhees showed the possibility of inverse ripening depending on the morphology and size of the precipitates, but stabilization against ripening has not been observed for a many-particle system. Wang *et al*.^30,31^ reported inverse ripening in a 2D system with many precipitates. However, again, the effect was found to be local and strongly dependent on the size and arrangement of the neighbouring precipitates. In contrast to these observations, the chemo-mechanical coupling effect, (viz,. the composition dependence of elastic constants) is shown to result in a more generic inverse ripening phenomenon. This is because in this scenario the chemo-mechanical coupling results in a strained concentration filed around each individual precipitate that is, to the first-order, independent of the size of precipitate^15,16^. Figure 6 clearly shows the strong solute depletion around the precipitates, that only exists when a chemo-mechanical coupling effect is present. This makes the current inverse ripening as a global feature of the system, when other sources of stress are absent. It is notable that the conditional inverse ripening, as proposed in previous works, in the absence of chemo-mechanical coupling (*κ* = 0) was not observed for *δ*′ precipitates^17^.

In order to understand the current results in the context of previous works on the *δ*′ precipitation kinetics, critical differences between the previous and current experiments should be considered. The most important of these differences is that the total aging time in the current experiment was limited to 13 hours (at 200 °C), which resulted in the *maximum* average radius of 7.5 nm for *δ*′ precipitates. These values are much smaller than those reported in most of the previous studies on the Al-Li system; the *minimum* average precipitate radii studied were about 20 nm after 25 hours of aging at 225 °C (Al-2.1 wt.%Li) by Pletcher *et al*.^13^ and about 17 nm after 50 hours of aging at 200 °C (Al-2.4 wt.%Li) studied by Mahalingam *et al*.^44^. Using the SAXS technique, Tsao *et al*.^10^ observed small precipitates with an average radius of 10 nm for an Al-9.7 at.% Li alloy aged at 180 °C up to 3.5 hours. However, again, similar to the current study the evolution of the average radius and size distribution of the precipitates could not be explained consistently using the previous theories.

If the aging period is extended as it is in most previous studies, it has been often observed that large *δ*′ precipitates form and grow on the structural defects, for example, dislocations. Indeed, for longer aging periods, large *δ*′ precipitates were found to decorate dislocations and PFZs^42,45^. Since the significance of chemo-mechanical coupling, and therefore inverse ripening, strictly depends on the stress state around the precipitates^15,16^, one should think of dislocations as a major disturbance to the inverse ripening mechanism; when the stress state around the precipitate is disturbed, the mechanically driven fluxes responsible for inverse ripening change as the elastic energy landscape will be largely modified in the vicinity of the dislocations. Furthermore, the mechanism of precipitation and growth can change to a great extent owing to pipe diffusion along the dislocations^46^ and variation of the composition (saturation) in the PFZs. In the current study, although the dissolution of the precipitates at the dislocations was clearly observed after 11 hours of aging, formation of large precipitates at the dislocations and next to the PFZs was not observed during the limited period of the experiment.

The stress state around precipitates might also change owing to structural modifications at the interface. Previous theoretical studies noted that coherency loss can restore conventional ripening^17^ from inverse ripening. Similarly, if the interface energy increases over time, the driving force for conventional ripening increases and the inverse ripening will be hindered.

## Conclusion

*In-situ* TEM experiments and phase-field simulations of *δ*′ precipitation in an Al–7.8 at.%Li alloy were performed up to 13 hours of aging at 200 °C. The TEM observations reveal the evolution of precipitates accompanied with a loss of precipitates fraction, attributed to the loss in Li content, and formation of a rather narrow size distribution of the precipitates. The loss of Li content could be due to generation of dislocations and existence of free surfaces in the simulation set-up. The amount of this loss was quantified by measuring the number density and volume fraction of precipitates over time, and was emulated considering a sink in the simulation set-up. In the presence of chemo-mechanical coupling, which arises due to the dependence of elastic constants on the Li content in the matrix solid solution, the evolution of the precipitates in terms of number density, average precipitate radius and size distribution could be explained consistently. The results indicate that an inverse ripening mechanism could be active in this size range of the *δ*′ precipitates, as predicted in recent theoretical studies^16,17^. This mechanisms is a general feature of the alloy system and it is responsible for the development of the narrow size distributions observed in the *in-situ* experiments. The comparison to the previous studies implies that the mechanism of inverse ripening could be mostly relevant to the earlier stages of ripening as it is possibly disturbed by different sources of stress in the system. In particular, dislocations can play a critical role in restoring conventional ripening of the precipitates. The current study is the first attempt to realize the significance of chemo-mechanical coupling on the precipitation kinetics. Further investigations in this direction are necessary to establish a general understanding of this kind of coupling and its effect on the microstructure evolution.

## Methods

### Experimental Procedure

A 1 mm-thick Al-Li sheet (exact composition: 7.84 at% (2.14 wt.%) Li, 0.04 at.% Si, 4 × 10^−4^ at.% Cu and (balance) Al) was prepared by vacuum induction melting followed by solution treatments and hot/cold rolling. The sheet was cut into 10 mm × 10 mm × 1 mm pieces, solution treated at 580 °C for 30 minutes and water-quenched. After conventional thinning to 100 *μ*m, 3 mm-disk TEM specimens were jet polished in a recirculating chiller environment. *In-situ* TEM experiments up to 13 hours and high-resolution (HR) analysis were carried out using JEOL JEM-2100, 2100 F and Gatan 652 double tilt heating holder to investigate the precipitate evolution at 200 °C. The specimen temperature was ramped up to the target temperature in a minute and held for analyses while maintaining temperature control within 1 K. Micrographs of the pointed area were taken every hour at the target temperature. The specimen is clamped on its edge but can expand as there is a hole (500 *μ*m dia.) at the centre, which is very large compared to the specimen thickness (approx. 100 nm). Further details of the analyses are given in the Supplementary Materials.

### Modelling and Simulation

Multi-phase-field method has shown a great potential in studying microstructure evolution in solid state^47–50^. Here, this method was applied for studying precipitation growth and ripening without and with a chemo-mechanical coupling effect. The details of the multi-phase-field modelling of precipitation with a chemo-mechanical effect^16,17^ and the numerical benchmarks of the chemo-mechanical problems^51^ were presented and discussed in earlier works. In the current simulations, each precipitate as well as the matrix phase are indexed with an unique phase-field parameter. Hence a coalescence of the precipitates is prohibited. A generalized time-dependent Ginzburg-Landau equation is employed to track the evolution of non-conserved phase-field parameters^52^. Both chemical and elastic energy contributions to the precipitation are taken into account, recovering the kinetic Gibbs-Thompson relation

3 $$\frac{V}{L}=\gamma K+{\rm{\Delta }}{G}^{{\rm{chem}}}+{\rm{\Delta }}{G}^{{\rm{elast}}}$$

where *V* is the local velocity of interface, *L* is interface mobility, *γ* is interface energy, and *K* is the local curvature. The chemical driving force Δ*G*^chem^ is taken from a parabolic free energy function to be proportional to the local deviation from the equilibrium interface composition, while the elastic energy density difference between the two phases is taken as the elastic driving force Δ*G*^elast^. A Reuss homogenization is applied at the interface, and during the simulation, mechanical equilibrium was maintained by solving $\nabla {{\boldsymbol{\sigma }}}_{ij}=\overrightarrow{0}$, where *σ*_*ij*_ is the stress tensor.

The simulations are performed using the software *OpenPhase*^53,54^. Initial time step and grid size are chosen as 0.25 *s* and 1 *nm* while the box size is 300 × 300 × 100 grid cells, which resembles the observed volume in the *in-situ* experiment. Periodic boundary conditions are applied. The interface energy^55^ is 0.014 Jm^−2^. The diffusion coefficient of Li in an Al matrix^56^ at 200 °C (473.15 K) is 1.2 × 10^−18^ m^2^ s^−1^. The elastic constants *C*_*ij*_ of FCC *α*-Al and L1_2_ *δ*′ as well as the coupling value *κ* for the matrix, following Eq. (1), and the misfit strain ε^*^ due to the precipitation^4^ are listed in Table 1.

**Table 1:** Elastic constants *C*_*ij*_ and coupling factors *κ*_*ij*_ of matrix and precipitate phase.

Elastic properties	*α*-Al (matrix)				*δ*′ (precipitate)			
	*C*_11_	*C*_12_	*C*_44_	κ	*C*_11_	*C*_12_	*C*_44_	*ε* ^*^
[GPa], at.%^−1^, %	107.1	62.9	28.9	0.05	139.8	33.7	40.7	−0.0975

In this study, the coupling factor *κ* = 0.05 at.%^−1^ is chosen by adopting the best match to the evolution of number density and average precipitate size in the experiment. This is found to be in the range of the ab-initio values applied in previous studies^16,17^. The size of the precipitates in the initial microstructure is obtained from the *in-situ* experiment after 9 hours and the spatial distribution of the precipitates is random. Though in a random initialization some precipitates might overlap, they have different phase-field indexes that results in an interface between them (no neck formation and coalescence). Since a continuous loss of Li content was evidenced in the experiments, a Li sink was placed at the top and bottom surface of the calculation domain to account for this loss of solute atoms. This could result from the segregation of Li to the dislocations or other defects (e.g,. surfaces), as discussed in above. The blue layers in Fig. 1 represent the Li sink. The strength of this sink has been adopted to match the evolution of the precipitate volume fraction *f*_*p*_ determined in the experiment:

4 $${f}_{p}=\frac{\sum _{i\mathrm{=1}}^{n}\frac{4}{3}\pi {r}_{i}^{3}}{{n}_{x}\cdot {n}_{y}\cdot {n}_{z}}\mathrm{.}$$

Here, *n* is the considered number of precipitates, *r* is the individual precipitate radius, and *n*_*x*_*i*s are the system dimensions. Assuming stoichiometric properties for *δ*′ (fixed precipitate concentration *c*_*p*_ = 25 at.%), the average equilibrium matrix concentration ${c}_{m}^{eq}$ for the initial time step can be calculated using the lever rule as

5 $${c}_{m}^{eq}=\frac{{c}_{tot}^{init}-{f}_{p}\cdot {c}_{p}}{1-{f}_{p}}$$

with ${c}_{tot}^{init}$ as the total Li concentration. The total matrix concentration at the final state ${c}_{tot}^{final}$ can be calculated as

6 $${c}_{tot}^{final}=(1-{f}_{p})\cdot {c}_{m}^{eq}+{f}_{p}\cdot {c}_{p}$$

where a constant equilibrium matrix concentration is assumed. Thus the difference between the initial and final concentrations ${c}_{tot}^{final}-{c}_{tot}^{init}$, which is a function of the volume fraction of the precipitates, gives the measured loss of Li content, which is exactly met in the simulations by inserting a sink.

## Supplementary information

Supp Info

## Data Availability

Both experimental data and simulation codes are available per reasonable request.

## References

[1] Williams JC, Starke EA (2003). Progress in structural materials for aerospace systems. Acta Materialia.

[2] Miller WS (2000). Recent development in aluminium alloys for the automotive industry. Mater. Sci. Eng. A.

[3] Wang SC, Starink MJ (2005). Precipitates and intermetallic phases in precipitation hardening Al-Cu-Mg-(Li) based alloys. Int. Mater. Rev..

[4] Prasad, N. E., Gokhale, A. A. & Wanhill, R. J. H. Aluminum-lithium alloys, processing, properties and applications (Butterworth-Heinemann, Oxford, 2014).

[5] Lifshitz I, Slyozov V (1961). The kinetics of precipitation from supersaturated solid solutions. J. Phys. Chem. Solids.

[6] Wagner C (1961). Theorie der Alterung von Niederschlägen durch Umlösen (Ostwald-Reifung). Zeitschrift für Elektrochemie.

[7] Rylands LM, Wilkes DMJ, Rainforth WM, Jones H (1994). Coarsening of precipitates and dispersoids in alurninium alloy matrices: a consolidation of the available experimental data. J. Materials Science.

[8] Kalogeridis A, Pesicka J, Nembach E (1999). On the increase of the precipitated volume fraction during Ostwald ripening, exemplified for aluminium-lithium alloys. Materials Science And Engineering A - Structural Materials Properties Microstructure And Processing.

[9] Ardell AJ (1997). Temporal behavior of the number density of particles during Ostwald ripening. Mater. Sci. Eng..

[10] Tsao C-S, Chen C-Y, Kuo T-Y, Lin T-L, Yu M-S (2003). Size distribution and coarsening kinetics of *δ*′ precipitates in Al–Li alloys considering temperature and concentration dependence. Mater. Sci. Eng. A.

[11] Davies C, Nash P, Stevens RN (1980). The effect of volume fraction of precipitate on ostwald ripening. Acta metallurgica.

[12] Ardell A (1972). The effect of volume fraction on particle coarsening: theoretical considerations. Acta metallurgica.

[13] Pletcher BA, Wang K-G, Glicksman ME (2012). Ostwald ripening in Al-Li alloys: A test of theory. International Journal of Materials Research.

[14] Wang KG, Glicksman ME, Rajan K (2004). Modeling and simulation for phase coarsening: A comparison with experiment. Phys. Rev. E.

[15] Darvishi Kamachali R, Borukhovich E, Shchyglo O, Steinbach I (2013). Solutal gradients in strained equilibrium. Philos. Mag. Lett..

[16] Darvishi Kamachali R, Schwarze C (2017). Inverse ripening and rearrangement of precipitates under chemomechanical coupling. Comput. Mater. Sci..

[17] Schwarze C, Gupta A, Hickel T, Darvishi Kamachali R (2017). Phase-field study of ripening and rearrangement of precipitates under chemomechanical coupling. Phys. Rev. B.

[18] Larché FC, Cahn JW (1982). Overview 25: The effect of self-stress on diffusion in solids. Acta Metall..

[19] Larché FC, Cahn JW (1985). Overview 41: The interactions of compositions and stress in crystalline solids. Acta Metall..

[20] Khachaturyan, A. G. Theory of structural transformations in solids (Courier Corporation, 2013).

[21] Cahn JW (1962). On spinodal decomposition in cubic crystals. Acta metallurgica.

[22] Wang Y, Khachaturyan A (1995). Shape instability during precipitate growth in coherent solids. Acta metallurgica et materialia.

[23] Löchte L, Gitt A, Gottstein G, Hurtado I (2000). Simulation of the evolution of gp zones in al–cu alloys: an extended cahn–hilliard approach. Acta Materialia.

[24] Voorhees P, Johnson WC (2004). The thermodynamics of elastically stressed crystals. Solid State Physics-Advances Res. Appl..

[25] Shi, S., Markmann, J. & Weissmüller, J. Verifying larché–cahn elasticity, a milestone of 20th-century thermodynamics. *Proc. Natl. Acad. Sci*. 201809355 (2018). 10.1073/pnas.1809355115 PMC6205470 30291190

[26] Taga A, Vitos L, Johansson B, Grimvall G (2005). Ab initio calculation of the elastic properties of al_1−*x*_li_*x*_ (*x* ≤ 0.20) random alloys. Phys. Rev. B.

[27] Darvishi Kamachali R, Borukhovich N, Hatcher E, Steinbach I (2014). DFT-supported phase-field study on the effect of mechanically driven fluxes in *Ni*_4_*Ti*_3_ precipitation. Model. Simul. Mater. Sci. Eng..

[28] Johnson W (1984). On the elastic stabilization of precipitates against coarsening under applied load. Acta Metall..

[29] Johnson WC, Voorhees P, Zupon D (1989). The effects of elastic stress on the kinetics of ostwald ripening: the two-particle problem. Metall. Transactions A.

[30] Wang Y, Chen L-Q, Khachaturyan A (1992). Particle translational motion and reverse coarsening phenomena in multiparticle systems induced by a long-range elastic interaction. Phys. Rev. B.

[31] Wang Y, Chen L-Q, Khachaturyan A (1993). Kinetics of strain-induced morphological transformation in cubic alloys with a miscibility gap. Acta Metall. et Materialia.

[32] Su C-H, Voorhees P (1996). The dynamics of precipitate evolution in elastically stressed solids—i. inverse coarsening. Acta materialia.

[33] Su C, Voorhees P (1996). The dynamics of precipitate evolution in elastically stressed solids—ii. particle alignment. Acta materialia.

[34] Al-Kassab T, Menand A, Chambreland S, Hassen P (1992). The early stages of decomposition of al-li alloys. Surf. Sci.

[35] Hono K, Babu SS, Okano R, T. S (1992). Atom probe study of early stage phase decomposition in an al-7.8at.% li alloy. Acta Metall. et Materialia.

[36] Schmitz G, Hono K, Haasen P (1994). High resolution electron microscopy of the early decomposition stage of Al-Li alloys. Acta Metallurgica et Materialia.

[37] Kobayashi S, Nakai K, Ohmori Y (2012). Analysis of ordering process in an al-li alloy by a newly developed method of degree of order determination using high-resolution transmission electron micrographs. Metall. Mater. Transactions A.

[38] Neibecker P (2017). L1_2_ ordering and *δ*′ precipitation in Al-Cu-Li. Sci. Reports.

[39] Yoshi-yama T, Hasebe K, Mannami M (1968). Al3Li superlattice in Al-4.5wt.% Li alloy. J. Phys. Soc. Jpn..

[40] Baumann S, Williams D (1985). Experimental observations on the nucleation and growth of *δ*′ (Al3Li) in dilute al-li alloys. Metall. Transactions A.

[41] Cottrell AH, Bilby BA (1949). Dislocation theory of yielding and strain aging of iron. Proc. Phys. Soc. Sect. A.

[42] Baumann SF, Williams DB (1985). Effects of capillarity and coherency on *δ*′ (Al3Li) precipitation in dilute al-li alloys at low undercoolings. Acta Metall..

[43] Miyazaki T, Seki K, Doi M, Kozakai T (1986). Stability bifurcations in the coarsening of precipitates in elastically constrained systems. Mater. Sci. Eng..

[44] Mahalingam K, Gu B, Liedl G, Sanders T (1987). Coarsening of *δ*′(Al3Li) precipitates in binary al-li alloys. Acta metallurgica.

[45] Williams D, Edington J (1975). The precipitation of *δ*′ (Al3Li) in dilute aluminium–lithium alloys. Met. Sci..

[46] Legros M, Dehm G, Arzt E, Balk TJ (2008). Observation of giant diffusivity along dislocation cores. Science.

[47] Darvishi Kamachali, R. Grain boundary motion in polycrystalline materials. Ph.D. thesis, Ruhr-Universität Bochum, Bochum, Germany (2013).

[48] Darvishi Kamachali R, Hua J, Steinbach I, Hartmaier A (2010). Multiscale simulations on the grain growth process in nanostructured materials. Int. J. Mater. Res..

[49] Schwarze C, Darvishi Kamachali R, Steinbach I (2016). Phase-field study of zener drag and pinning of cylindrical particles in polycrystalline materials. Acta Materialia.

[50] Darvishi Kamachali R, Kim S, Steinbach I (2015). Texture evolution in deformed AZ31 magnesium sheets: Experiments and phase-field study. Comput. Mater. Sci..

[51] Darvishi Kamachali, R. *et al*. Numerical Benchmark of Phase-Field Simulations with Elastic Strains: Precipitation in the Presence of Chemo-Mechanical Coupling. *Comput. Mater. Sci*. 541–553 (2018).

[52] Steinbach I, Pezzolla F (1999). A generalized field method for multiphase transformations using interface fields. Phys. D: Nonlinear Phenom..

[53] Interdisciplinary Centre for Advanced Materials Simulation, Ruhr-University Bochum. OpenPhase, http://www.openphase.de/.

[54] Tegeler M (2017). Parallel multiphase field simulations with openphase. Comput. Phys. Commun..

[55] Baumann SF, Williams DB (1984). A new method for the determination of the precipitate-matrix interfacial energy. Scripta Metall..

[56] Skrotzki, B. & Murken, J. On the effect of stress on nucleation, growth and coarsening of precipitates in age-hardenable aluminium alloys. in: K. V. Jata (Ed.), Light Weight Alloys for Aerospace Applications VI, The Minerals, Metals & Materials Society, Warrendale, P. A., USA, 2001, pp. 51–62 (2001).

