# Supplementary material for: First Evidence for Mechanism of Inverse Ripening from In-situ TEM and Phase-Field Study of δ′ Precipitation in an Al-Li Alloy
Source: Sci Rep. 2019 Mar 8;9:3981. doi: 10.1038/s41598-019-40685-5 (PMC6408572; doi:10.1038/s41598-019-40685-5)
Supplement: Supplementary file 1 — Supp Info [file 41598_2019_40685_MOESM1_ESM.pdf]

Supplementary Information for
First Evidence for Mechanism of Inverse Ripening from In-situ TEM and Phase-Field Study of δ' Precipitation in an Al-Li Alloy

Jiwon Park, Reza Darvishi Kamachali, Sung-Dae Kim, Su-Hyeon Kim, Chang-Seok Oh, Christian Schwarze, Ingo Steinbach

This file includes:

1. Detailed imaging techniques
2. Details about evolution of precipitates
3. Development of precipitate-free microstructure and dislocations

1 Detailed imaging techniques

In Fig. S1 (a) showing TEM bright-field image of as-quenched specimen before heat treatment, only few dislocations were in visible region. While the bright-field image does not show noticeable contrast from δ' phase, diffraction pattern in S1 (b) clearly indicates superstructure reflection from δ' having $L1_2$ structure. Figure S1 (c) and (d) are disordered FCC and ordered $L1_2$ structures, and their diffraction patterns obtained from the as-quenched specimen. Due to the small difference in lattice parameters between two phases, diffraction patterns overlap each other as marked in red and yellow dots. Overall distribution of the δ' precipitates was investigated through dark-field imaging from $\{100\}$ diffractions, which attributed to $L1_2$ superstructure. In comparison between bright-field and dark-field imaging as shown in Fig. S2, the precipitates are more distinguishable in the dark-field micrograph. In assessing individual precipitate, however, spatial resolution of dark-field imaging is not sufficient to measure precise size of the precipitate especially in the initial stage of aging.

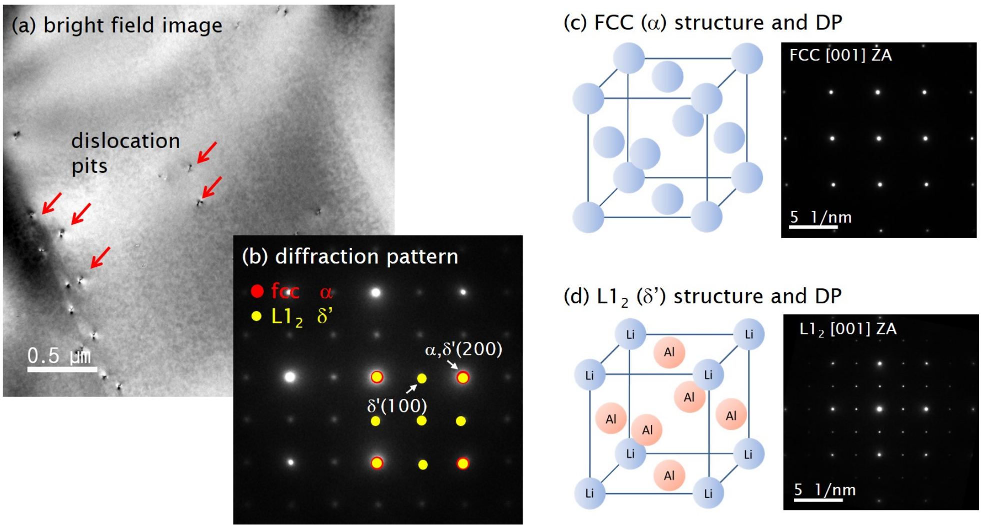

Fig. S1: (a) TEM bright-field image showing low dislocation density before heat treatment. Dislocation pits are marked with arrows. (b) Diffraction pattern corresponding to (a). (c) FCC(α) structure and its diffraction pattern (d) L1$_2$(δ') structure and its diffraction pattern

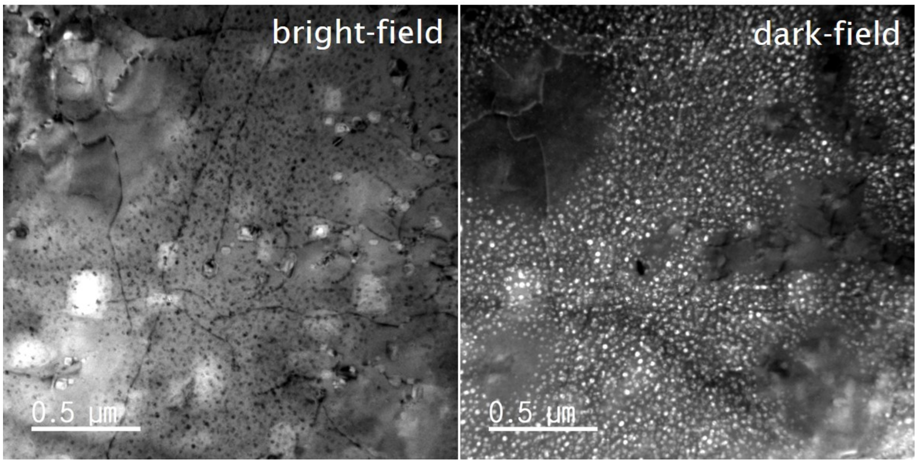

Fig. S 2: Comparison between bright-field and dark-field imaging from the same region

As the size of a precipitate in the early stage of aging is measured in few nanometers, HR images and their fourer-filtered images were used to determine the precipitate radius precisely. In Fig. S3, an acquired HR image is fast fourer transformed (FFT) to select {100} and {110} spots corresponding to the ordered L1$_2$ structure indicated in the orange circles. The inverse-FFT images in the yellow-boxed regions are depicted in the clear lattice images when the region attributes to the ordered L1$_2$ structure, i.e. δ' precipitate. On the other hand, the disordered FCC lattice is diffuse due to zero intensities of {100} and {110} structure factors.

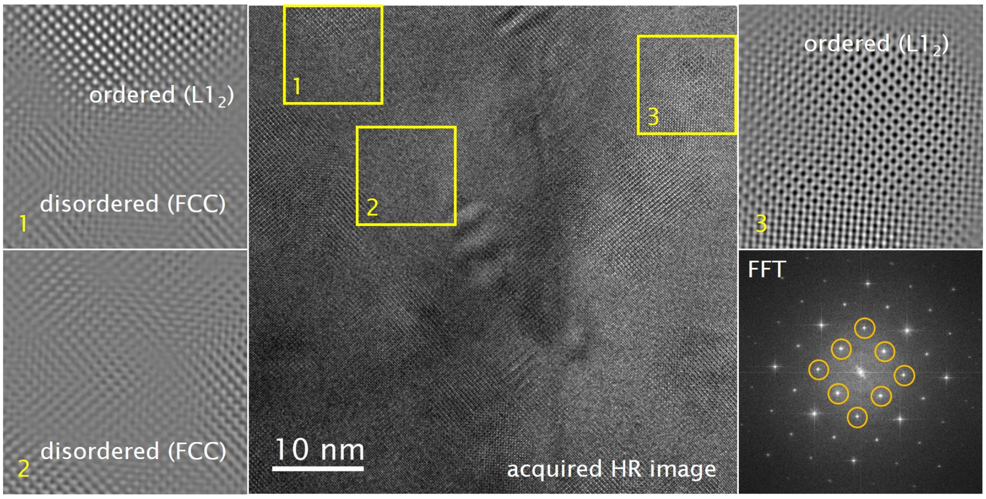

Fig. S 3: HR image, its FFT and inverse-FFT images showing clear $L1_2$ and diffuse FCC lattices.

2 Details on evolution of precipitates

Precipitate size distribution: The change in experimental size distribution during the in-situ experiment are shown in Fig. S4 (a) below, and also visible for instance in TEM micrographs Fig. 5 (a)-(c) in the paper. These data were used to plot Fig. 7 in the paper.

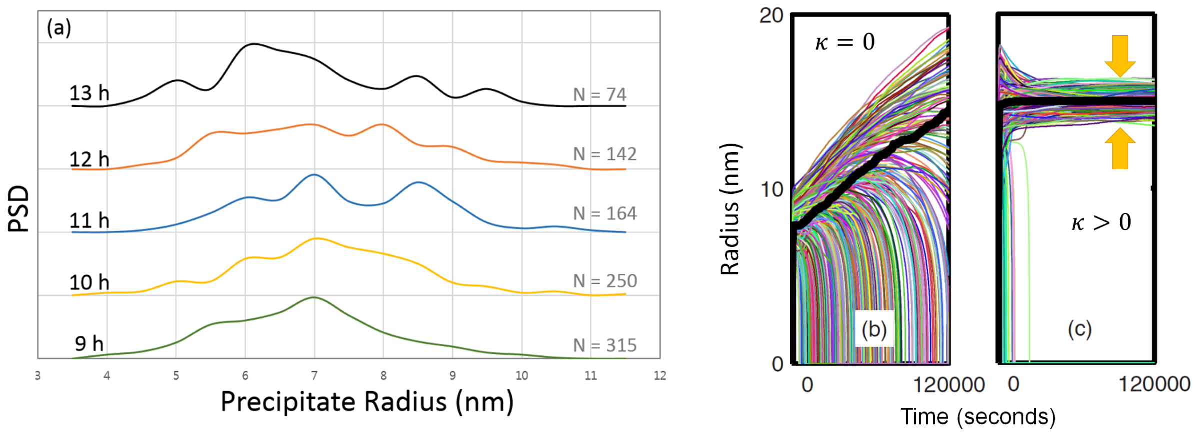

Fig. S 4: (a) Precipitate size distribution changing during in-situ TEM observation. N indicates the number of precipitates measured in each time-frame. Evolution of individual precipitates in a large phase-field simulation for cases (b) without and (c) with a chemo-mechanical coupling. While in a conventional ripening the smaller precipitates shrink and disappear, in the presence of chemo-mechanical coupling inverse ripening occurs. The arrows show the range of precipitates radii in an inverse ripening process [See Schwarze et al, Physical Review B 95 174101, 2017].

In the simulations, the general broadening of the size distribution in the presence of the chemo-mechanical coupling is due to the elastic interaction among the precipitates that was also observed in extended theoretical studies presented previously. To demonstrate this, Fig. S4 (b)–(c) below shows the evolution of individual precipitates over time without and with a chemo-mechanical coupling [See Schwarze et al, Physical Review B 95 174101, **2017**]. Here the yellow arrows indicate the range of precipitate sizes. Even after a long time, the precipitates clearly avoid the single sharp size (being a delta function) that is due to the elastic interaction among them.

Temporal Evolution of precipitates in the simulations: In Figure S5 2D cross-sections of the diffuse phase-field interfaces and corresponding concentration maps are plotted over time for both simulation cases ($\kappa = 0$ and $\kappa = 0.05$). The initial size of the precipitates are extracted from the in-situ TEM experiment. In the random initialization step some of precipitates meet as it is visible in the 9h microstructure. These precipitates, however, are not able to coalesce as each individual precipitate has a unique phase-field parameter (multi-phase-field method).

It is observed that in the presence of the chemo-mechanical coupling the concentration of the matrix is clearly higher. Also a strong solute depletion next to the precipitates is observed that is a characteristic feature of the chemo-mechanical effect as demonstrated in previous studies [Darvishi Kamachali, Schwarze, Comp Mat Sci 130 292-296, **2017**]. 3D presentations of the simulation box (Fig. 6) clearly show that, Compared to the conventional ripening, smaller precipitates shrink slower in the presence of chemo-mechanical coupling, that is due to the inverse ripening phenomenon. In this scenario, the overall shrinkage is due to the Li sink present in the simulation, while in the absent of the chemo-mechanical effect conventional ripening was observed.

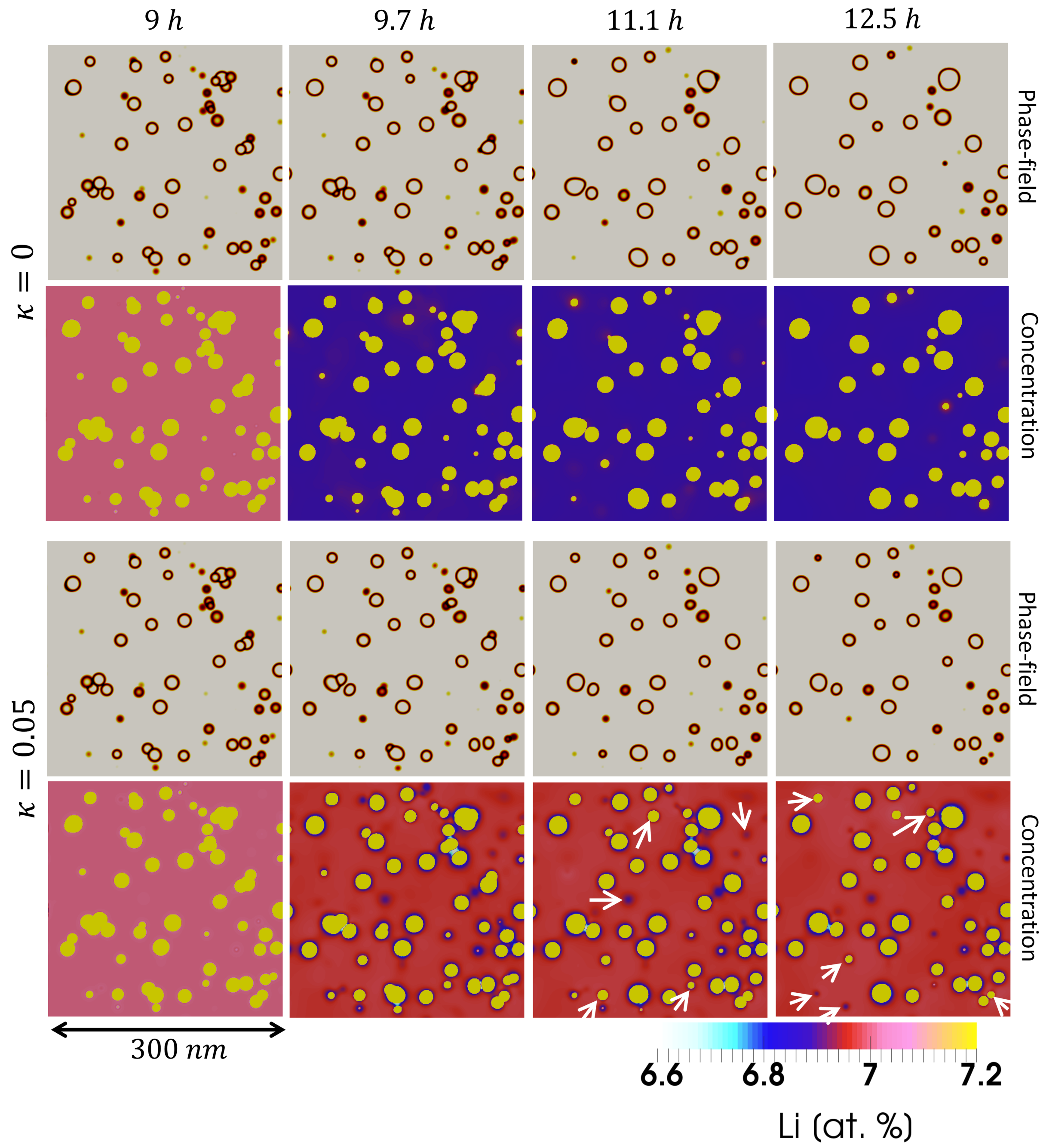

Fig. S5: 2D cross-section of diffuse phase-field interfaces and concentration field over time. Starting from initial microstructure and Li sink, the evolution of the precipitates without and with the chemo-mechanical coupling are different.

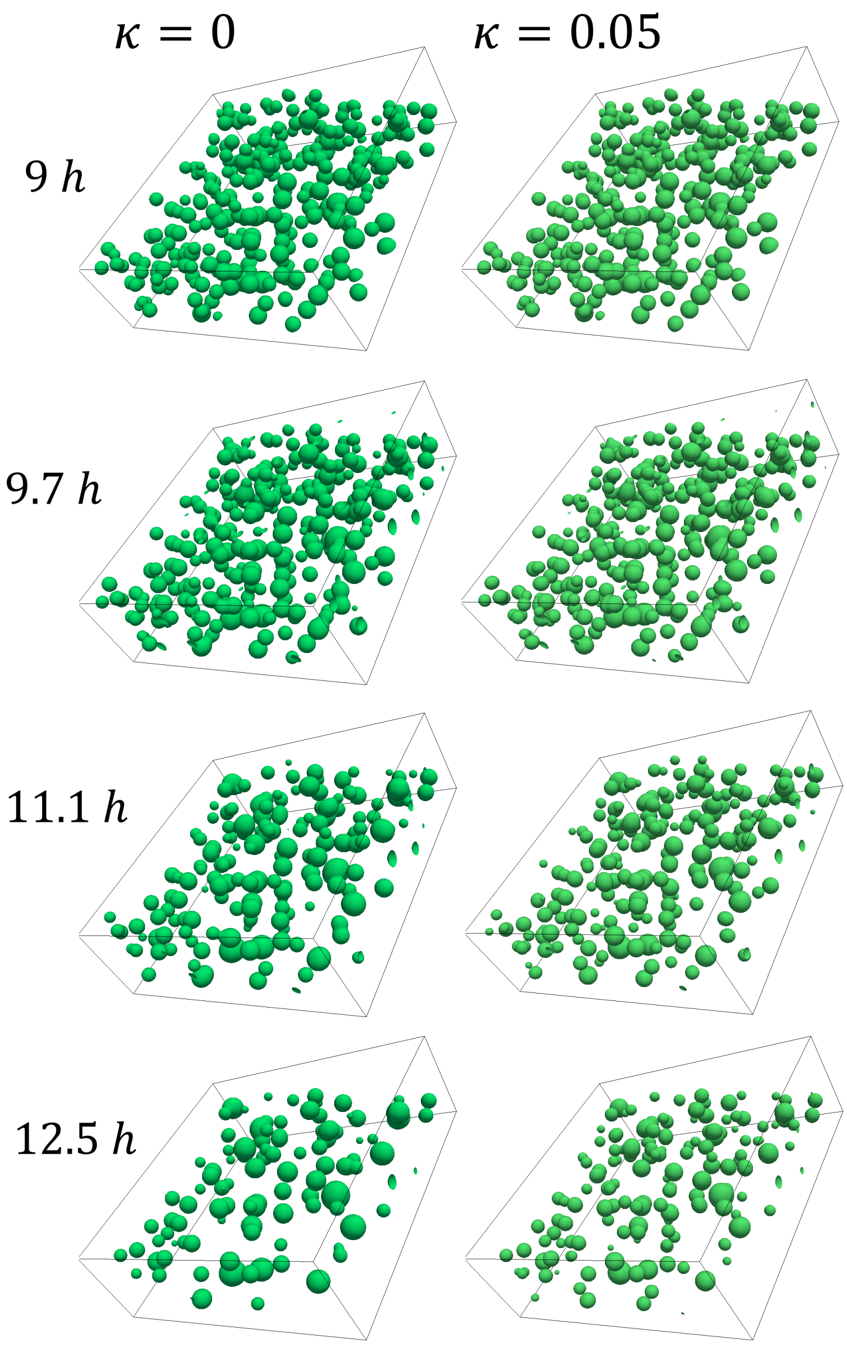

Fig. S 6: 3D snapshots of the simulation box at different times. Left: $\kappa = 0$, Right $\kappa = 0.05$.

3 Development of precipitate-free microstructure and dislocations

The TEM observations confirm that a precipitate-free zone form. Figure S7 below shows the evolution of precipitate-free microstructure during in-situ aging. The yellow box indicates the same area, which is in top-left corner of the Figure 4 in the paper. We found that precipitate-free zone was developed closely associated with dislocations during in-situ aging. The thin-specimen configuration (thickness ∼100 nm) in the in-situ TEM experiment affect on dislocation generation. Once dislocations are generated from the pre-existing defects, surface roughness, etc., they are trapped inside the specimen due to the absence of dislocation sink such as grain boundary. Recrystallization and grain growth, which occur during heat treatment of the bulk metallic sample, are inhibited in the thin specimen without grain boundary.

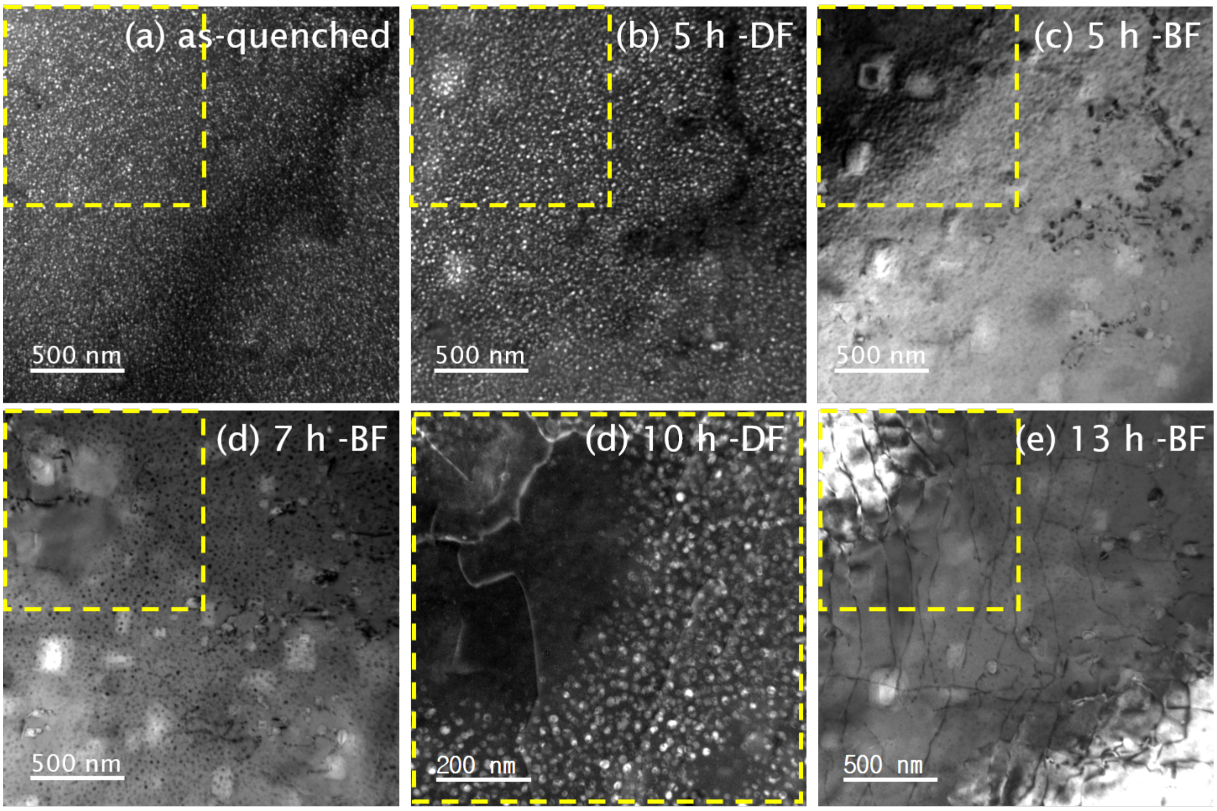

Fig. S7: Evolution of precipitate-free microstructure during in-situ aging. (a) as-quenched state showing evenly distributed precipitates (b) dark-field and (e) bright field at 5 h aging (d) precipitate-free zone starts growing from 7 h to (d) 10 h and (e) 13 h. The yellow boxes indicate the same area.
